# Herbal Prescription SH003 Alleviates Docetaxel-Induced Neuropathic Pain in C57BL/6 Mice

**DOI:** 10.1155/2021/4120334

**Published:** 2021-08-10

**Authors:** Kangwook Lee, Jin Mo Ku, Yu-Jeong Choi, Hyun Ha Hwang, Miso Jeong, Yun-Gyeong Kim, Min Jeong Kim, Seong-Gyu Ko

**Affiliations:** ^1^Institute of Safety and Effectiveness Evaluation for Korean Medicine, Seoul 02453, Republic of Korea; ^2^Department of Science in Korean Medicine, Graduate School, Kyung Hee University, Seoul 02453, Republic of Korea; ^3^Department of Preventive Medicine, College of Korean Medicine, Kyung Hee University, Seoul 02453, Republic of Korea

## Abstract

Docetaxel-based therapy has been applied to kill cancers including lung and breast cancers but frequently causes peripheral neuropathy such as mechanical allodynia. Lack of effective drugs for chemotherapy-induced peripheral neuropathy (CIPN) treatment leads us to find novel drugs. Here, we investigated whether and how novel anticancer herbal prescription SH003 alleviates mechanical allodynia in mouse model of docetaxel-induced neuropathic pain. Docetaxel-induced mechanical allodynia was evaluated using von Frey filaments. Nerve damage and degeneration in paw skin of mice were investigated by immunofluorescence staining. Neuroinflammation markers in bloodstream, lumbar (L4-L6) spinal cord, and sciatic nerves were examined by ELISA or western blot analysis. Docetaxel (15.277 mg/kg) was intravenously injected into the tail vein of C57BL/6 mice, and mechanical allodynia was followed up. SH003 (557.569 mg/kg) was orally administered at least 60 min before the mechanical allodynia test, and von Frey test was performed twice. Docetaxel injection induced mechanical allodynia, and SH003 administration restored withdrawal threshold. Meanwhile, degeneration of intraepidermal nerve fibers (IENF) was observed in docetaxel-treated mice, but SH003 treatment suppressed it. Moreover, docetaxel injection increased levels of TNF-*α* and IL-6 in plasma and expressions of phospho-NF-*κ*B and phospho-STAT3 in both of lumbar spinal cord and sciatic nerves, while SH003 treatment inhibited those changes. Taken together, it is worth noting that TNF-*α* and IL-6 in plasma and phospho-NF-*κ*B and phospho-STAT3 in spinal cord and sciatic nerves are putative biomarkers of docetaxel-induced peripheral neuropathy (DIPN) in mouse models. In addition, we suggest that SH003 would be beneficial for alleviation of docetaxel-induced neuropathic pain.

## 1. Introduction

Cancer patients receive chemotherapy to kill malignant tumors and improve survival rate whereas it unfortunately causes severe side effects [1, 2]. CIPN is one of the painful side effects occurring in approximately 30–70% of patients and characterized by damaged peripheral neurons [3–6]. The main symptoms include pain, muscle weakness, and muscle spasms, resulting in a decrease in the quality of life [7]. Taxanes (paclitaxel and docetaxel) are useful anticancer drugs but cause CIPN [8]. Docetaxel is one of the cytotoxic anticancer drugs and exhibits an anticancer effect by binding to tubulin, resulting in impairment of microtubule homeostasis and mitotic arrest [9]. US Food and Drug Administration (FDA) approved docetaxel as an anticancer drug against multiple types of cancers including non-small-cell lung and breast cancers [10, 11]. Of note, docetaxel is associated with acute pain syndrome in several cancer patients [12–17]. Development of painful symptoms by DIPN may lead to discontinuation of cancer treatment regardless of therapeutic benefit of chemotherapy [18–20]. Until now, there have been no effective therapeutic options for DIPN in cancer patients [21]. Thus, this drives us to find novel medicines for treatment of DIPN.

Cancer chemotherapy typically damages the distal sensory neurons of hands or feet with severe pain [22]. Nonclinical studies have demonstrated that peripheral nervous system tissues, which are mainly damaged in CIPN model, include sciatic nerves, lumbar spinal cord, and dorsal root ganglion (DRG) [23, 24]. While the mechanism underlying CIPN is still unclear, several CIPN studies have demonstrated that NF-*κ*B has been suggested to be one of the readouts for neuropathic pain [8, 25]. It was reported that activation of NF-*κ*B is associated with spinal cord and sciatic nerve injury in rodent model [26]. Moreover, inhibition of NF-*κ*B pathway can ameliorate chronic pain in neuropathic pain rodent model. Besides NF-*κ*B, STAT3 pathway has also been suggested to be involved in neuropathic pain. It has been reported that paclitaxel treatment induces neuropathic pain with increased expression of phosphorylated JAK2 and STAT3 [27]. Furthermore, another study demonstrated that chemotherapeutic agent bortezomib-induced mechanical allodynia is associated with STAT3 activation in DRG [28]. Moreover, TNF-*α*-mediated activation of STAT3 plays a critical role in CIPN [29]. Therefore, activation of NF-*κ*B and STAT3 can be regarded as a biomarker for CIPN. However, there is no evidence to support the fact that those biomarkers are associated with DIPN.

Novel herbal prescription SH003 is the traditional Chinese medicine (TCM) theory-based anticancer drugs composed of *Astragalus membranaceus*, *Angelica gigas*, and *Trichosanthes kirilowii* Maximowicz. The anticancer effect of SH003 against breast, lung, and prostate cancer has been demonstrated through several *in vitro* and *in vivo* studies [30–38]. The safety of SH003 was proved by phase I clinical study for solid tumors, and phase II clinical study for wild-type EGFR lung cancer patients is in progress [39–41]. Meanwhile, phase I/II clinical study of SH003 combined with docetaxel for breast and lung cancer patients is also in progress (NCT01755923, https://clinicaltrials.gov). While it is a crucial point that TCM-based herbal medicines and supplements are the representative alternative treatment for improving health problems including cancer-related side effects [42–46], we needed to investigate whether novel herbal medicine SH003 alleviates docetaxel-mediated adverse effects in both nonclinical and clinical studies.

The purpose of the present study was to evaluate the mitigative effect of SH003 on DIPN in C57BL/6 mice. Intravenous injection of docetaxel decreased the withdrawal threshold of von Frey filament compared to Control group whereas oral administration of SH003 relieved this symptom. ELISA analysis showed that levels of TNF-*α* and IL-6 in plasma were upregulated by docetaxel injection while SH003 treatment revered it. Docetaxel-induced upregulation of phospho-NF-*κ*B and phospho-STAT3 expression in sciatic nerve and spinal cord was inhibited by SH003 treatment. Histological analysis of mouse foot pad skin indicated that administration of SH003 relieved docetaxel-induced damage on peripheral nerve fibers. Therefore, the present study suggests that SH003 is applicable to alleviate DIPN.

## 2. Materials and Methods

### 2.1. SH003 and Docetaxel

SH003 powder was prepared as described previously. In brief, *Astragalus membranaceus* (333 g), *Angelica gigas* (333 g), and *Trichosanthes kirilowii* Maximowicz (333 g) were mixed and then extracted with 10 times volume of 30% ethanol at 100°C for 3 h. This process was performed 2 times. The extract was dried at reduced pressure (40 Torr) at 60°C for 18 h. Dried SH003 was stored at −20°C. Docetaxel was purchased from Sigma-Aldrich (#01885–25MG-F, St. Louis, MO, USA). Docetaxel was dissolved in DMSO and stored at −20°C.

### 2.2. Determination of Drug Dose

No-observed-adverse-effect level (NOAEL) of SH003 determined by phase I clinical trials was 4,800 mg/day [41]. Maximum tolerable dose (MTD) of docetaxel in cancer patients was 75 mg/m^2^ [40]. Animal equivalent dose was calculated by the following equation [47]:

(1) $$\begin{array}{c} \text{human equivalent dose }\left(\frac{\text{mg}}{\text{kg}}\right)=\text{animal dose }\left(\frac{\text{mg}}{\text{kg}}\right)\times \left(\frac{\text{weight animal }\left(\text{kg}\right)}{\text{weight human }\left(\text{kg}\right)}\right)\left(1-0.75\right). \end{array}$$

Human and mice weights are 65 kg and 0.02 kg, respectively. Since there is an individual difference in metabolic rate, exponent for body surface area is 0.75. Thus, animal equivalent dose of SH003 and docetaxel was determined as 557.569 mg/kg and 15.277 mg/kg, respectively.

### 2.3. Docetaxel-Induced Neuropathy Mouse Model and Experiment Schedule

**Figure 1:**
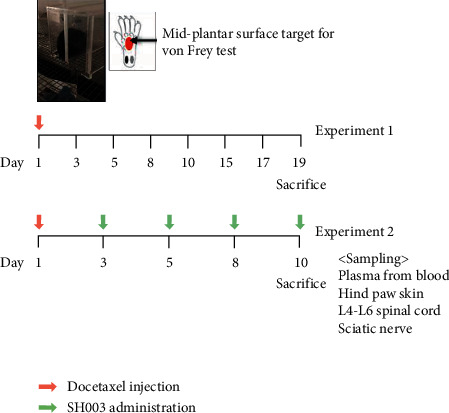
Timelines of the *in vivo* experimental design.

All procedures in animal experiments were approved by Kyung Hee University Institutional Animal Care and Use Committee (KHU-IACUC; KHSASP-19-322). C57BL/6N mice (5 weeks old) were purchased from Nara Biotech (Seoul, South Korea). Mice were housed under constant temperature (24 ± 2°C) with light-dark cycle, and food and water were freely available. Two experiments were performed. The timelines of experiment “1” and experiment “2” are shown in Figure 1.

In experiment “1,” mice were randomly divided into different treatment groups as follows: Control (*n* = 6) and Docetaxel (*n* = 6). Before injection of docetaxel on the 1^st^ day, baseline withdrawal threshold in each mouse was determined. Mice were treated with a single intravenous injection of docetaxel to model DIPN, and neuropathic symptom was followed up.

In experiment “2,” mice were randomly divided into different groups as follows: “Control” (*n* = 5), “Docetaxel” (*n* = 5), “Docetaxel + SH003” (*n* = 5). After determination of baseline withdrawal threshold by von Frey test on the 1^st^ day, mice in “Docetaxel” and “Docetaxel + SH003” were intravenously injected with 50 *μ*L docetaxel while mice in “Control” were injected with the same volume of DMSO, which did not exceed 5 mL/kg according to guidelines [48]. SH003 was orally administered at least 60 min before the performance of mechanical allodynia test. SH003 administration and von Frey filament test were performed 2-3 times a week. At the end of the experiment, mice were euthanized by 100% carbon dioxide in chamber by 30–70%/min filling rate, followed by cervical dislocation. The hind paw skin of mice was collected for staining of IENF. For evaluation of the inflammatory damage on peripheral neurons, lumbar (L4-L6) spinal cord and both left and right sciatic nerves were collected.

### 2.4. Measurement of Mechanical Allodynia

Mechanical allodynia was evaluated using von Frey filaments (JD-SI-11F, Jeungdo B and P, Seoul, South Korea) according to standard operating procedure from the Jackson Laboratory mouse neurobehavioral phenotyping facility. In brief, the room atmosphere was maintained with constant temperature (24 ± 2°C), humidity (50 ± 20%), and lighting (400–500 lux) without noise. Prior to the start of von Frey test, mice were individually placed on stainless steel mesh floor with acrylic cover (L^*∗*^W^*∗*^H= 10^*∗*^8^*∗*^7 cm) and left without disturbance for a minimum of 30 min to acclimate. The midplantar surface of left hind paw of mice was poked with filament (0.4 g) for 3 seconds until it bends, and each application was presented at least 3 times. If there was no response with the starting filament (0.4 g), it was changed to the next highest filament (0.6 g) in the series and continuously moved through the series in order until a withdrawal response was observed or the 2 g filament was presented. If there was response with the starting filament, it was moved to the next lowest filament in the series until a withdrawal response was not observed or the 0.02 g filament was presented. Licking and shaking of the affected paw were determined as the withdrawal responses. The whole procedure from acclimation to determination of withdrawal responses was repeated once again. Mechanical allodynia threshold values from two independent experiments were recorded and averaged in each group.

### 2.5. Immunofluorescence Staining of IENF

The hind paw skin was isolated from all mice and immediately fixed with 4% paraformaldehyde for 6 h. Fixed skin samples were dehydrated using 30% sucrose solution for 16 h. For the cryosection, dehydrated tissues were embedded in OCT compound and frozen at liquid nitrogen. Frozen sections of 20 *μ*m were stained with anti-PGP9.5 antibody (1 : 800, ab8189, Abcam, Cambridge, UK) and IgG secondary antibody conjugated with Alexa Fluor 488 (1 : 2,000, A28175, Invitrogen Co., Carlsbad, CA, USA). The images were acquired using Zeiss LSM 5 PASCAL confocal laser scanning microscope system (Carl Zeiss AG, Oberkochen, Germany) at a magnification of 10x.

### 2.6. Enzyme-Linked Immunosorbent Assay (ELISA)

Levels of inflammatory cytokines TNF-*ɑ* and IL-6 were assessed using a DuoSet ELISA kit (555268 and 555240, respectively, BD Biosciences, San Diego, USA) according to the manufacturer's instructions. In brief, the whole blood was collected from left ventricle and transferred to EDTA tube (367835, BD Biosciences, San Diego, USA). After centrifugation at 2,000 rpm for 10 min, plasma from blood was prepared and stored in a deep freezer at −80°C until use. 96-well plates were coated with capture antibody in ELISA coating buffer and incubated overnight at 4°C. The plates were then washed with wash buffer and subsequently blocked with assay diluent at RT for 1 h. Diluted standard and plasma samples were added to plates and incubated at RT for 2 h. Detection antibody and streptavidin-conjugated horseradish peroxidase were added to the plates, followed by incubation at RT for 1 h. Tetramethylbenzidine substrate was added and incubated at RT for 0.5 h. The reaction was ended by adding stop buffer. The optical density was measured at 450 nm on an automated ELISA reader (Versa Max, Molecular Devices, CA, USA).

### 2.7. Western Blot Analysis

Total protein from lumbar (L4-L6) spinal cord and sciatic nerves were extracted using RIPA buffer (R2002, Biosesang, Gyeonggi-do, South Korea) with protease and phosphatase inhibitors. The same amount of proteins was quantified by Bradford protein assay (Bio-Rad, Hercules, CA, USA). The proteins were separated by 8 or 15% SDS-PAGE, followed by transfer to nitrocellulose membranes. After blocking with blocking buffer including 1% (w/v) skim milk and 1% (w/v) BSA at RT for 1 hour, the membrane was incubated with anti-NF-κB (#8242), anti-phospho-NF-κB (#3033), anti-STAT3 (#4904), anti-phospho-STAT3 (#9145), and anti-GAPDH (#5174) at 4°C for 16–24 h. All antibodies were purchased from Cell Signaling Technology (Beverly, MA, USA). Horseradish peroxidase- (HRP-) conjugated secondary IgG antibodies (Calbiochem, San Diego, CA, USA) were further incubated with the membrane at RT for 1 h. An enhanced chemiluminescence kit (DG-WP100, DoGen, Seoul, South Korea) was used for detection of HRP signal.

### 2.8. Statistical Analysis

Statistical analysis was performed using Prism (GraphPad, San Diego, CA, USA). The differences of means between the groups were analyzed by one-way or two-way ANOVA using Tukey's or Sidak's multiple comparisons test, respectively. *P* value < 0.05 means statistically significant difference. Results were represented as mean ± standard deviation (SD) or standard error of the mean (SEM).

## 3. Results and Discussion

### 3.1. Intravenous Injection of DIPN in C57BL/6 Mice

**Figure 2:**
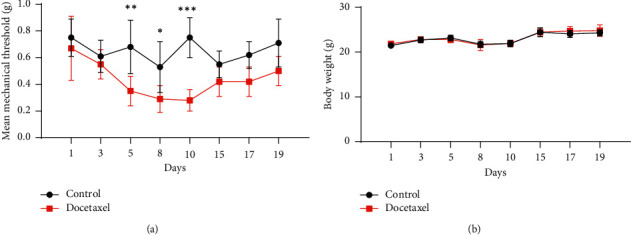
Docetaxel injection induced mechanical allodynia in C57BL/6 mice. The mice were divided into “Control” (*n* = 6) and “Docetaxel” (*n* = 6). Docetaxel was intravenously injected via tail vein of C57BL/6 mice on the 1^st^ day, and mechanical allodynia threshold by von Frey filament was followed up. Mean mechanical threshold (a) and body weight (b). The differences of means between the groups were analyzed by two-way ANOVA using Sidak's multiple comparisons test (^*∗*^*P* < 0.05, ^*∗∗*^*P* < 0.01, and ^*∗∗∗*^*P* < 0.001; Docetaxel *vs*. Control).

Docetaxel is a well-known anticancer drug with severe side effects including CIPN with pain in the hands and feet [49,50]. The present study was performed to model DIPN. Docetaxel was intravenously injected into mice tail vein, and mechanical allodynia threshold by von Frey test was followed up. On the 5^th^ day, mechanical withdrawal threshold of Docetaxel group began to be lower than that of Control group (Figure 2(a)). On the 10^th^ day, the biggest difference of mechanical allodynia threshold between Control group and Docetaxel group occurred, while there were no differences after the 15^th^ day (Figure 2(a)). Drug treatment did not affect body weights of mice until 19 days (Figure 2(b)). Therefore, we determined to perform further efficacy study until the 10^th^ day. It is common for taxane-based adjuvant chemotherapy to induce acute pain syndrome [51]. In fact, the incidence of taxane acute pain syndrome is more common with docetaxel than paclitaxel whereas docetaxel triggers less peripheral neuropathy than paclitaxel [16, 52]. Docetaxel-mediated pain flare occurs within 4–5 days after docetaxel chemotherapy and lasts about 4 days [52]. Consistent with clinical findings, *in vivo* model for docetaxel-related acute pain syndrome was successfully developed by a single intravenous injection of docetaxel at MTD.

### 3.2. Oral Administration of SH003 Mitigated Docetaxel-Induced Mechanical Allodynia at Hind Paws of C57BL/6 Mice

**Figure 3:**
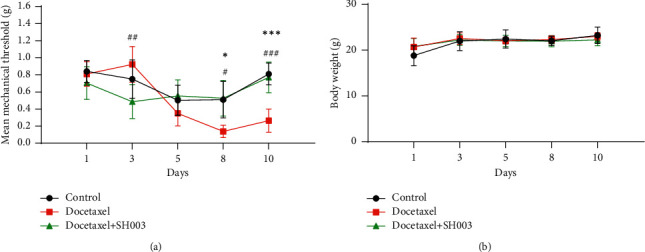
Effect of SH003 administration on docetaxel-induced mechanical allodynia in C57BL/6 mice. The mice were divided into Control (*n* = 5), Docetaxel (*n* = 5), and Docetaxel + SH003 (*n* = 5) groups. Docetaxel was intravenously injected via tail vein of C57BL/6 mice on the 1^st^ day, and mechanical allodynia threshold by von Frey filament was followed up. From 3^rd^ day, SH003 was orally administered at least 60 min before the performance of mechanical allodynia test. Mean mechanical threshold (a) and body weight (b). Data are presented as mean ± SEM. The differences of means between the groups were analyzed by two-way ANOVA using Sidak's multiple comparisons test (^*∗*^*P* < 0.05, Docetaxel *vs*. Control; ^*∗∗∗*^*P* < 0.001, Docetaxel *vs*. Control; ^#^*P* < 0.05, Docetaxel + SH003 *vs*. Docetaxel; ^###^*P* < 0.001, Docetaxel + SH003 vs. Docetaxel).

Next, we further examined whether SH003 mitigates docetaxel-induced mechanical allodynia. Although mechanical threshold of “Docetaxel” was higher than that of “Docetaxel + SH003” on the 3^rd^ day, the threshold value of two groups was not statistically different with “Control.” While docetaxel treatment induced mechanical allodynia, SH003 treatment relieved it on the 8^th^ and 10^th^ day (Figure 3(a)). Drug treatment did not affect body weights of mice until 10 days (Figure 3(b)). The data showed that SH003 administration mitigated DIPN. SH003 is a TCM-based novel herbal mixture for treating several malignant cancers including breast, prostate, and lung cancers. Anticancer effect and mode of action have been clearly demonstrated by numerous nonclinical studies [30–38]. In fact, conventional chemodrugs have adverse side effects because of their cytotoxicity to healthy cells as well as cancer cells regardless of therapeutic benefit. Our previous study reported that SH003 is safe in rats according to toxicity studies with Good Laboratory Practice (GLP) regulations [36]. Oral administration of SH003 at various doses (500; 1,000; 2,000 mg/kg) did not show any problem in mortality, food intake, haematological value, organ weight, histopathology, etc. In sum, we suggest that SH003 is safe and effective herbal medicine for treatment of DIPN as well as cancer.

### 3.3. SH003 Administration Inhibited Docetaxel-Induced Reduction of IENF at Hind Paws of C57BL/6 Mice

**Figure 4:**
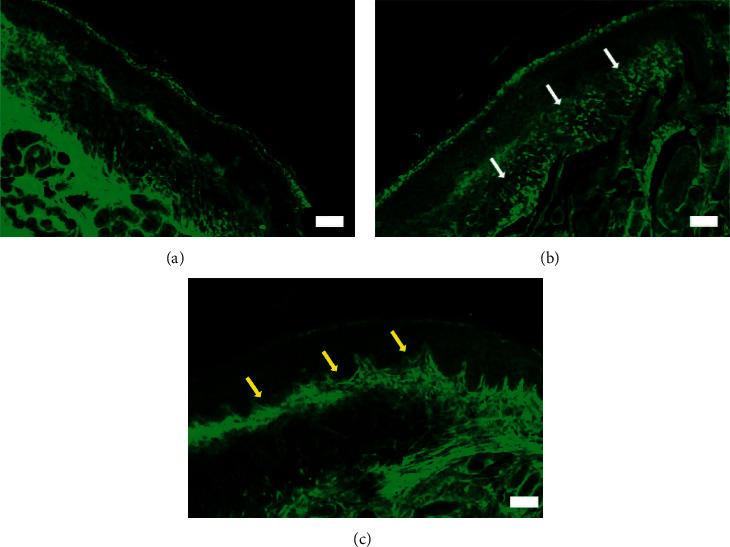
Effect of SH003 administration on degeneration of IENF induced by docetaxel. The hind paw skins were isolated and prepared for immunofluorescence staining of IENF in Control (a), Docetaxel (b), and Docetaxel + SH003 (c) groups. The representative images of PGP 9.5-labeled IENF in paw skins were acquired using Zeiss LSM5 PASCAL confocal laser scanning microscope system. White scale bar indicates 50 *μ*m (magnification: 10x). White and yellow arrows indicate the degenerated IENF and stratum lucidum layer, respectively.

IENF are bare nerve endings within junction between dermis and epidermis and play a role in transmission of peripheral pain [53, 54]. Clinically, chemotherapy damages distal IENF, resulting in loss of IENF in hands and feet in patients with CIPN. [55–59]. In the rodent CIPN model, degeneration of IENF in paw skin is detected although the contribution of IENF loss to the pathobiology of CIPN is not fully elucidated [60–62]. Of note, chemotherapeutic drugs including paclitaxel and oxaliplatin induce loss of IENF with severe pain in rodent model while prevention of IENF loss inhibits neuropathic pain [53, 55, 63]. Thus, the reduction of IENF loss could be regarded as one of therapeutic markers in CIPN model. We further investigated the therapeutic effect of SH003 on IENF degeneration in mice paw skin. Compared to Control, IENF seem to be fragmented and degenerated (white arrow) in docetaxel-treated mice, whose morphological changes indicate axonal degeneration (Figures 4(a) and 4(b)). SH003 treatment decreased docetaxel-induced histopathological changes whereas a layer (yellow arrow) was thickened (Figure 4(c)). This layer located in the mid-epidermis is stratum lucidum containing dead keratinocytes and is present in the palms and soles [64]. The thickness of this layer is regulated by the rate of mitosis of the epidermal cells [65]. Stratum lucidum layer contains a large amount of eleidin which is known to protect skins by blocking infiltration of water [66]. However, the role of stratum lucidum in neuropathic pain is unknown yet. Although further studies are still needed to investigate the possible role of components in stratum lucidum in DIPN model, our data suggest that histological change in stratum lucidum is one of the therapeutic markers for SH003 on DIPN.

### 3.4. Oral Administration of SH003 Inhibited Docetaxel-Induced Production of Proinflammatory Cytokines in Bloodstream of C57BL/6 Mice

**Figure 5:**
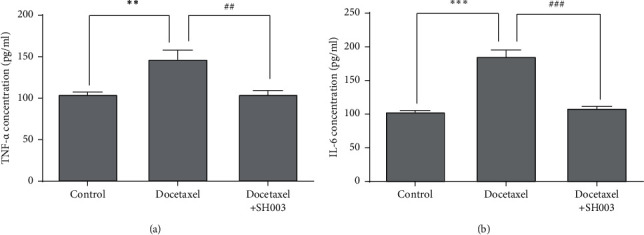
Effect of SH003 administration on the expression of TNF-*α* and IL-6 in plasma of DIPN mice. Plasma sample was prepared, and levels of inflammatory cytokines TNF-*α* (a) and IL-6 (b) were assessed using a DuoSet ELISA kit according to the manufacturer's instructions. Data are presented as mean ± SD of the mean (*n* = 5 for each group). The differences of means between the groups were analyzed by one-way ANOVA using Tukey's post hoc test (^*∗∗*^*P* < 0.001, Docetaxel *vs*. Control; ^*∗∗∗*^*P* < 0.001, Docetaxel *vs*. Control; ^##^*P* < 0.01, Docetaxel + SH003 *vs*. Docetaxel; ^###^*P* < 0.001, Docetaxel + SH003 *vs*. Docetaxel).

In CIPN model, pain mediators including inflammatory cytokines are increased in peripheral nerve spinal cord and sciatic nerves [67]. *In vivo* studies demonstrated that levels of TNF-*α* and IL-6 are elevated in CIPN animal model [68, 69]. It was also demonstrated that neutralizing antibodies against TNF-*α* or IL-6 reduce chemotherapy-induced allodynia [69, 70]. The present study investigated whether SH003 reduces TNF-*α* and IL-6 in DIPN model. Docetaxel injection increased levels of TNF-*α* and IL-6 in plasma sample (Figures 5(a) and 5(b)). The present data showed that DIPN is associated with inflammatory neuropathy [8]. In SH003 group, the inflammatory level increased by docetaxel was reduced as much as Control group (Figures 5(a) and 5(b)). We also demonstrated that SH003 inhibition of DIPN is mediated by relieving inflammation.

### 3.5. Oral Administration of SH003 Inhibited Docetaxel-Induced Phosphorylation of NF-*κ*B and STAT3 in Both L4-L6 Spinal Cord and Sciatic Nerve of C57BL/6 Mice

**Figure 6:**
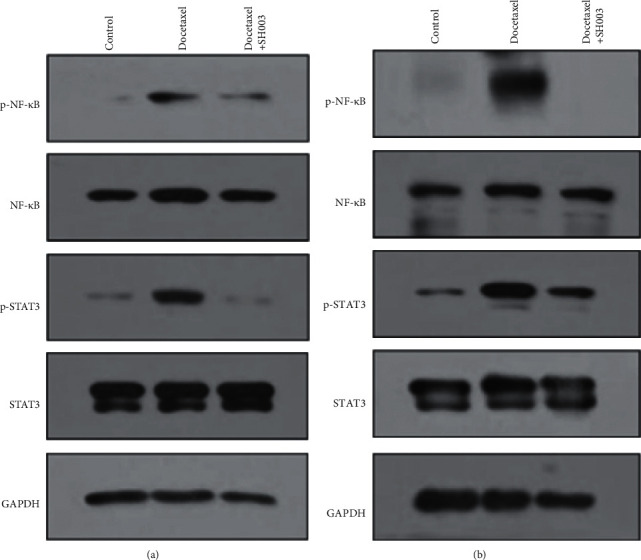
Effect of SH003 administration on the expression of NF-*κ*B and STAT3 in lumbar (L4-L6) spinal cord and sciatic nerves of DIPN mice. At the end of the experiment, lumbar (L4-L6) spinal cord and sciatic nerves were isolated. Total proteins were extracted and separated by SDS-PAGE. Expression of NF-*κ*B and STAT3 from spinal cord (a) and sciatic nerves (b) was analyzed by western blotting. GAPDH was used as internal marker.

NF-*κ*B and STAT3 would be one of the readouts for CIPN [8, 25–29]. Moreover, TNF-*α* and IL-6 are involved in the activation of NF-*κ*B and STAT3 in neuroinflammation [71–73]. Thus, we further examined whether SH003 treatment inhibits NF-*κ*B and STAT3 in both of lumbar spinal cord and sciatic nerves. Docetaxel treatment increased the expression of phospho-NF-*κ*B and phospho-STAT3 in both L4-L6 spinal cords and sciatic nerves whereas SH003 treatment reversed it (Figures 6(a) and 6(b)). The present study suggested that activation of NF-*κ*B and STAT3 is a putative biomarker of DIPN while further studies are necessary to decipher how they are involved in the development and progression of DIPN. Moreover, SH003 inhibition of NF-*κ*B and STAT3 shown in our data would be a hint for SH003 application to other peripheral neuropathic diseases. Deregulation of NF-*κ*B and STAT3 appears to be involved in the progression of neuropathic pain in diabetes [74–77]. Alcoholic neuropathy is tightly linked to NF-*κ*B deregulation [78, 79]. NF-*κ*B deregulation is likely to be related to dysimmune neuropathies such as acute Guillain–Barré syndrome [80]. Therefore, SH003 may be applicable for treating other neuropathic diseases related to the deregulation of NF-*κ*B and STAT3.

## 4. Conclusions

Single injection of docetaxel at MTD can develop acute pain syndrome model in C57BL/6 mice. Moreover, activation of NF-*κ*B and STAT3 in sciatic nerves and L4-L6 spinal cords and upregulation of TNF-*α* or IL-6 in bloodstream may be biomarkers of DIPN in mouse model. However, further studies are needed to investigate the role of those putative biomarkers in the development and progression of DIPN. Furthermore, we suggest that SH003 administration is able to relieve docetaxel-induced neuropathic pain and this efficacy needs to be monitored in current clinical studies.

## Data Availability

All datasets used and/or analyzed during the current study are available from the corresponding author on reasonable request.
